# Caring for Computers: The Hidden Work of Clinical Nurses during the Introduction of Health Information Systems in a Teaching Hospital in Taiwan

**DOI:** 10.3390/nursrep11010011

**Published:** 2021-02-13

**Authors:** Feng-Tzu Huang

**Affiliations:** Liberal Arts Center, Department of Nursing, Da-Yeh University, Changhua 51591, Taiwan; fzh@mail.dyu.edu.tw

**Keywords:** computerization, duality of technology, health information systems, hidden work, nurses’ experience

## Abstract

Implementing health information systems for enhancing patient care and management occurs worldwide. Discovering how nurses, as important system end-users, experience technology-reliant clinical practice involved focus groups (*n* = 25) and in-depth individual interviews with nurses (*n* = 4) and informatics staff (*n* = 3) in a major Taiwanese medical center. This qualitative study explores the unintended effects of these systems on nurses’ role and clinical practice. First, nurses’ additional role caring for computer devices supporting patient care involves highly-demanding invisible effort, especially when tackling system malfunctions affecting patients with urgent conditions. Second, nurses are resourceful in developing solutions to protect patients during unexpected technical malfunctions. Third, troubleshooting using telephone technical support as the first resort is problematic. It is argued that computerization requires nurses to care for co-clients: patients and computers. Managing technical malfunctions is an unintended consequence for nurses, reflecting the hidden work required by new technology.

## 1. Introduction

The discipline of health informatics arose during the 1970s [1] as an umbrella title covering medical, nursing, dental, and pharmacy informatics. It is generally defined as computer sciences applied for collecting, managing, using, and sharing information to support healthcare delivery and promote health [2,3,4]. Furthermore, the term “health information systems” (HIS) is used to mean “a set of components and procedures organized with the objective of generating information which will improve healthcare management decisions at all levels of the health system … integrate data collection, processing, reporting and use of the information necessary for improving health service effectiveness and efficiency through better management at all levels (p. 3)” [5]. In this study, the term HIS indicates the aim of disseminating, communicating, and contributing to high-quality and efficient patient care [6], with an emphasis on improving action rather than gaining information [5,7].

Healthcare services are an information-intensive industry [8]. Information is significant at all levels of healthcare services, from patient and health unit management to policymakers, managers, and healthcare providers [9]. Computer technology and information science are now widely integrated within healthcare [10,11]. It is important to note that the practical development of HIS varies and terminologies are diverse. The term HIS is a general usage, which can involve, for example, nursing information systems, computerized nursing care plan systems, electronic patient records, computerized physician order entry, radiology information systems, and laboratory information systems. Globally, investment to facilitate HIS use has tended to increase, particularly in developing countries [6,12].

Initially, HIS development focused on administrative and financial activities related to healthcare service delivery, until the mid-1990s when HIS development began to focus on clinical management functions [8]. Thus, various types of HIS are widely applied in different clinical services, such as hospital management systems [2], medical patient record systems [13], and nursing information systems [14]. Abundant literature supports HIS applications and suggests that they have the potential to enhance quality of care and administrative efficiency, with various benefits claimed such as saving time spent on paper-based record keeping [15,16,17]; improving completeness of patient care records; facilitating quality assurance and research [18,19]; improving the accuracy of documentation; increasing the speed of information transfer [20,21]; facilitating the storage and retrieval of healthcare records [20,22], and providing up-to-date clinical information to help prevent errors [17,23]. In short, it is suggested that time-saving, completeness, accuracy, accessibility, and real-time usability are the main benefits of implementing HIS in healthcare management.

While the application of HIS in healthcare is widespread and looks promising, nurses, as the largest group of healthcare professionals, are the major HIS end-users [2]. Barnard [24,25] points out that nurses cannot use technology without being influenced by it. As governments worldwide have promoted the use of HIS, an important issue confronting nurses is how to utilize such information technologies in their daily clinical practice. Many studies have investigated the effectiveness and efficiency of adopting HIS in healthcare and management, but more knowledge is needed about how nurses respond to HIS-reliant clinical practice. Hence, this research asks the following question: What are the intended and unintended impacts of implementing HIS on the role of nurses and their clinical practice?

This qualitative case study explores how HIS have shaped nursing practice and care delivery and affected the nursing profession, and how nurses perceive HIS implementation. While extensively promoting HIS in the era of computerization, this article is mainly intended to examine the hidden work in nurses’ daily clinical practice rather than the technology itself. The significance of the case study is its exploration of practical situations affecting nurses while implementing HIS to enhance the quality of healthcare.

The following section reviews nurses’ experiences of computer use and explains the methods of collecting and analyzing data, followed by an examination of nurses’ HIS-reliant clinical work in a major medical center. The important issues about the unintended impact of HIS use on nurses’ role and practice will then be discussed, and conclusions presented.

## 2. Nurses’ Experiences of Computer Use

Nurses have experienced different changes arising from the utilization of HIS, with many studies finding positive changes along with some negative and contradictory impacts. In terms of positive perceptions, nursing information systems could act as a reference to aid memory and a learning tool to increase knowledge of patient care [26], leading to potential savings in time and resources [27] whilst encouraging accessible and efficient documentation [28,29]. Moreover, HIS adoption was perceived as helpful in understaffed units [30,31].

However, with HIS implementation, nurses perceived that time spent on the computer and on documentation had increased [32], which may affect the time available for direct patient care [33]. Nurses spent more time away from patients in order to complete the documentation and evaluate care plans, which became difficult since computer terminals were located in nursing stations rather than at the bedside [34]. Also, performance of nursing tasks was sometimes delayed because of time-consuming and difficult-to-use system functions [29,35,36]. Nurses in one study perceived that the computerized nursing care plan and nursing record were inflexible and inappropriate [30], and other studies have suggested that electronic patient records were unable to support individualized patient care [37,38]. Using the computerized nursing care plan was documentation-oriented rather than patient-care-oriented and could not reflect patients’ unique individual needs [27,30].

Furthermore, the impact of HIS standardization on nurses’ professional competence was noticed [34,39]. Nurses perceived that nursing information systems limited their discretion to make professional judgements [27,37], impaired critical thinking ability [29,40], lessened autonomy [41,42], undermined nurses’ ability to provide specialized individual care [30,38], and were unable to reflect “real nursing” [43], that is, nursing based on developing a one-to-one relationship with individual patients in order to assess their unique needs. Nurses thus perceived that computerization facilitated management control rather than improved nursing care [37].

Moreover, the negative influences of computer hardware and software problems and limitations on clinical practice were described in some studies. Nurses suggested that the computer system could not fully meet patients’ needs. Nurses also suggested their needs as end-users were not taken into account when planning the HIS, thereby failing to take into account the realities of nursing practice, such as the lack of computers in clinical areas [44,45], user-unfriendly interface designs [29,43,46], and system unreliability [38]. Thus, some nurses regarded the system design as inappropriate to nursing practice [43,45].

Some studies have attributed nurses’ dissatisfaction with nursing information systems to two reasons: “resistance to change” [34] and a need for more ”re-education and training” [28,47,48]. HIS development was driven by financial and administrative reasons, rather than the demands of professional nursing practice [2]. Darbyshire [43] criticized the fact that HIS development failed to consider the needs of end-users. Unsurprisingly, the development of HIS with inadequate support for professional nursing practice may hinder the level of acceptance by nurses [2,38].

Contradictory findings were noticed [49], with some studies finding positive changes and others negative results. These findings mainly involved qualitative studies with small-sized samples and self-reported data, leading to doubts about their generalizability. However, these findings provide vivid accounts from the nurses’ perspectives regarding the use of HIS in their practice. Transferability in qualitative research means that these studies could be applicable to other contexts. The perceived negative effects of HIS may have a profound impact on nurses themselves and nursing care. Furthermore, these studies were conducted in several countries worldwide and over different periods of time. Thus, the similar conclusions drawn from these studies cannot be ignored. They suggest that in certain circumstances, HIS may have negative impacts on nursing care and the nursing profession. Therefore, by focusing on a major Taiwanese medical center, this study aims to explore the intended and unintended consequences of HIS implementation on the role of nurses and their clinical practice.

## 3. The Study

### 3.1. The Case and Research Design

The aim of this study was to explore the perspectives and experiences of the registered nurses and informatics staff involved in HIS implementation and use. The importance is that the accounts from different perspectives could help construct a more comprehensive picture and an in-depth understanding of how these HIS were implemented and experienced.

The design of this qualitative, single-case study is based on the work of Yin [50]. It explores the impact of HIS on nurses in a major Taiwanese healthcare organization. The study case was a 1700-bed, private general teaching hospital in Taiwan. It is accredited as one of Taiwan’s 26 major medical centers [51] and has officially implemented HIS since 2007. The hospital provides general medical medicine, surgical medicine, intensive care, pediatrics, emergency, gynecology, and psychiatry from hospitalization to outpatient. Its outpatient clinics provide more than 60 subspecialties and serve about 5000 people daily. Its emergency department provides 24-h service for about 8000 patients monthly.

The findings presented in this paper were part of a larger study. The research design of the larger study was (1) conducting focus groups with registered nurses (*n* = 25), (2) elite interviews with nurse managers (*n* = 13), (3) elite interviews with informatics staff (*n* = 3), and (4) 47 h of nonparticipant observations in six wards of the case study hospital. During the later period of data collection time, some nurses unable or unwilling to participate in focus groups were interviewed in depth (*n* = 4). However, due to the paper length limit and the aim and scope, the findings of this paper, extracted from focus groups and in-depth interviews with nurses, and elite interviews with informatics staff, focus on the unintended effects of HIS on nurses’ role and clinical practice.

### 3.2. Methods

In terms of nurse participants, 12 wards were visited in order to recruit registered nurses. The recruitment process and methods included the following: (1) a designated date to orally introduce the study and give recruitment packages (information sheets, consent forms, and return slips) to all of the hospital’s nurses; (2) 10 min question times during day and evening shifts over two days in each of the 12 wards; (3) based on nurses’ return slips, individual face-to-face contact was made to discuss their willingness to participate, to answer any questions, to establish contact methods, to identify preferred times and locations for focus groups, and to build up trusting relationships. Five homogenous focus groups with front-line registered nurses were conducted in five wards units (*n* = 25). The participants formed the natural groups as they were colleagues. In addition, there were 4 individual, in-depth interviews with nurses.

A topic guide was used as a general guidance in each focus group and in-depth interview to encourage follow-up questions and discussions. Interviews began with asking nurses about how the hospital started to implement HIS and moved on to explore their experiences and viewpoints of using HIS in their daily nursing practice. Questions became more focused and specific as the discussions went on. Participants were also encouraged to lead the discussions into the areas that were important to them. The sessions lasted from 1 h 3 min to 1 h 49 min (focus groups) and from 50 min to 1 h 23 min (in-depth interviews), with responses being digitally recorded for later transcription, coding, and analysis.

By snowball sampling, elite interviews with informatics staff who had practical roles and experience of promoting the HIS in this medical center were carried out. The interviewed informatics staff included a division leader and two software engineers responsible for the HIS in the case study hospital (Table 1). The elite interviews lasted from 1 h 22 min to 2 h 15 min. All interviews were digitally recorded for later transcription, coding, and analysis. Each interview was guided by a topic guide asking the process and influence of HIS implementation from their personal experiences and future prospect of hospital development.

### 3.3. Participants

For the basic demographic data of the nurse participants, see Table 2 and Table 3. The participants constituted a reasonably mixed group of front-line nurses in terms of practice settings and nursing grades. However, in terms of age, years of experience, and educational background, the participants seemed to represent a younger group with Bachelor’s level of degrees and overall had used computers in their daily life.

### 3.4. Ethical Considerations

The researcher received the official approval from the Institutional Review Board (IRB) and layered permissions within the case study hospital to conduct this study. Confidentiality, anonymity, and voluntary participation were assured. In addition, as the participants of each focus group were colleagues, the confidentially and anonymity were potentially problematic [52]. Caveat emptor strategy by Tolich [53] was adopted to overcome these ethical issues. Participants were informed of the potential risks during the consent process and they were clearly stated in the information sheets. Rules of keeping the content confidential were mentioned prior to starting the discussions.

### 3.5. Data Analysis

The data were collected in Mandarin, and the digital records were transcribed. The interview data were analyzed employing thematic analysis [54]. The initial marginal codes were in Mandarin. Subsequently, formulating categories and reporting the results with quotes took place in English. Manual methods were chosen as these gave more control over the work [55], provided a greater degree of flexibility in keeping code memos, and allowed transcripts to be viewed holistically. The codes from the focus groups and interviews were compared and contrasted. This article focuses on those related to the perceived impacts of HIS on the role and practice of nurses. The findings are in three main groups: (1) invisible effort, (2) intangible coordination, and (3) troubleshooting.

### 3.6. Ensuring Rigour

From the perspective of the larger study, data source triangulation was adopted because participants from different levels of the organizational hierarchy as well as from the different professional groups were recruited, such as nurses as users, managers, and informatics staff. However, in this paper, findings from combing nurses’ accounts from focus groups and in-depth interviews also have advantages, such as being pragmatic, allowing participants’ perspectives to be compared and contrasted, and achieving data completeness [56]. The in-depth interviewees also discussed more sensitive topics and personal viewpoints when compared with focus group participants, which corresponded to Kaplowitz’s [57,58] findings. The perspectives and experiences of participants at different levels allowed the researcher to deeply explore the context of how HIS influenced nursing practice and compare the information obtained, thereby benefitting the data analysis process.

## 4. Results

### 4.1. Invisible Effort

Some participants perceived that their time spent on documentation had grown, which adversely affected the time available for patient care. Work was duplicated to comply with both paperwork and computer systems. A nurse described how her colleagues and she were busy between patients and computer systems:

*We need to take care of paper sheets printed, to take care of computer prescriptions, and then go to the bedside to proceed with these prescriptions … In the past we only needed to cope with paperwork, but now we spend time dealing with both computers and paperwork.*

(Nurse D5. Focus Group D. Pediatric Ward)

A member of the informatics staff said that computerization generates extra workloads for nurses:

*I think that computerization is beneficial to managers … But for nurses, it increases [their workload] as they need to do the data entry jobs.*

(Software Engineer IT2)

The three informatics staff interviewed all realized the above issue, with two (Division Leader IT1 and Software Engineer IT3) stressing that the way HIS fitted alongside nurses’ clinical work had been a key issue. Many participating nurses perceived that, although the operation of the HIS could enhance work efficiency, computer problems sometimes made them feel trapped and forced them to interrupt direct nursing care. As a result, they needed to take on the additional role of caring for computer devices, in order to successfully provide patient care. Moreover, the process of identifying and correcting HIS faults whilst simultaneously caring for acutely ill and medically unstable patients was very stressful. For many nursing participants, it was common to experience pressure from patients who were experiencing prolonged waiting times for treatment or discharge due to HIS failures or delays. For example, a nurse described how she was busy chasing the informatics staff whilst simultaneously facing stressed and distressed patients.

*It’s a very common situation that while I was processing patient discharge procedures, computers or printers were dead … I kept on contacting the informatics department, but they replied they were still investigating. [Meanwhile] patients kept on grumbling [about] why it took a long time to complete the discharge procedure.*

(Nurse D3. Focus Group D. Pediatric Ward)

Especially when talking about the occurrence of system crashes, four out of five focus groups had experienced unpredictable computer problems resulting from central operating system failure that meant all computers were unavailable; this was “miserable” according to one focus group:

*E3: System crashes are really miserable … When the central system fails … everyone ‘cries’ aloud.*

*M: Have you encountered it?*

*Everyone: Of course! [Rising tone]*

(Nurses. Focus Group E. Medical Ward)

Unsurprisingly, a nurse clearly indicated her perception about the pros and cons of the HIS:

*The HIS is convenient, but actually when the system crashes, it’s really a big trouble for you.*

(Nurse D1. Focus Group D. Pediatric Ward)

In this study, many nurses raised frequent printer malfunctions in focus group discussions. Four out of five groups reported daily printer problems. Other common problems mentioned were network disconnection, paper jams, and poor printing quality. A focus group described coping with printer problems as a time-consuming process:

*D1: Sometimes the computer has no problem, but the printer does … fixing it gives you a busy morning [and] cost you much time.*

*D3: It even costs you one or two hours.*

(Nurses. Focus Group D. Pediatric Ward)

Also, a nurse claimed that the important task of keeping printers in good condition had seemingly been devolved to nursing staff:

*How often is a printer supposed to have maintenance? We have to do it by ourselves.*

(Nurse B5. Focus Group B. Surgical Intensive Care Unit)

Concerning the way of identifying and correcting faults in the computer system, participating nurses said that in reality the official coping procedures were complex, lengthy, and were seldom used. While dealing simultaneously with computer problems and patient care, they either adopted quick workarounds or, if these failed, contacted informatics staff. The details of this process follow below.

### 4.2. Intangible Coordination

Compared with the lengthy, complex, official coping procedures for computer problems, many nurses explained their “work-arounds” that allow patient care to continue. Three out of five focus groups said that for patients with urgent conditions, during computer crashes they accepted doctors’ verbal orders for medications. However, without computerized prescriptions, the pharmacy could not dispense them. Hence, nurses adopted a quick, temporary solution—borrowing medications from other patients—until the system was restored:

*We encountered a computer crash during our evening shift, because of a system failure. There was a new patient admitted to our unit, and we wanted to give a blood transfusion and medications but we couldn’t. We could only wait for the system to be restored. The doctor could only give verbal orders for medications so we asked our colleagues whether their patients received the same medications and then borrowed from them urgently.*

(Nurse B2. Focus Group B. Surgical Intensive Care Unit)

Nevertheless, a nurse especially expressed her concerns about patients’ life because the prescriptions of blood transfusion therapy had been computerized. Once computer-related technological breakdown happens, the therapy of blood transfusion has no alternative, which was different from medications:

*So it is dangerous. For example, like a blood transfusion is completely computerized...If we don’t have the (printed) documents, we cannot request blood products. Medications can be borrowed, ‘cos other patients used the same medications. But for blood transfusion, it is dangerous. For emergency medications, they’re still manageable, ‘cos we’ve got the emergency trolley.*

(Nurse B5. Focus Group B. Surgical Intensive Care Unit)

For computer crashes or devices off-line, nurses contacted the informatics department. Focus group participants claimed that during the daytime, the informatics staff usually arrived at a ward within 30 min. However, troubleshooting sometimes took longer. If a computer problem could not be solved on-site, a substitute device was usually provided. However, if a computer problem involved other units, nurses needed to act as a coordinator to chase people. For instance, every focus group noted that because nurses’ system authority was limited, they needed to chase head nurses or doctors in order to re-print particular patient documents. A nurse described how she chased both the doctor and the informatics staff on duty in order to obtain the printed sheet for her patient’s blood transfusion:

*One night, my patient needed a blood transfusion treatment. The doctor prescribed on the computer from the duty room. [The printer in the nursing station malfunctioned]. Then I rang the doctor to check whether he had prescribed it. The doctor said he had. Because we nurses had no re-print authority, [we] can’t re-print the blood transfusion sheet, I [told] him that the printer didn’t print it out and I can’t print it out either, could you re-print it again for me? Then the doctor said he re-printed it, but still nothing printed it out. So I called the informatics department and they replied ‘How can it be possible?’ I asked if they had any method to re-print it, but they said they couldn’t. The IT staff suggested asking the doctor to prescribe it again using a different computer. So I needed to call the doctor again … It was embarrassing.*

(Nurse A5. Focus Group A. Medical Intensive Care Unit)

A similar situation concerned patients’ discharge procedures. Doctors often forgot to execute a compulsory step called “repertory of physician orders” from the doctor system. A nurse described how nurses chased the doctor to complete it in order to carry out a patient’s discharge procedures:

*Before we close patients’ accounts for the discharge procedures, the doctor must complete the repertory of physician orders. But the doctors often forget to do this step and leave the nursing station … I needed to phone him. Sometimes the doctor was busy [e.g., in theatre]. It would delay our discharge procedures … We can’t help him execute the step, because this belongs to the doctor’s order entry system. We need to keep phoning the doctor.*

(Nurse D1. Focus Group D. Pediatric Ward)

When talking about their experiences in implementing the HIS, nurses said positively that retrieving information easily from the database saved time searching through paper-based patient charts. Yet, the majority of focus groups agreed that the limited availability of computer devices caused problems with queues and borrowing computers. For example, a nurse said

*The day shift is more difficult, because many colleagues need to use the computer. Doctors and nursing practitioners need to prescribe and type their progress notes. We also need to use computers.*

(Nurse C3. Focus Group C. Surgical Ward)

All of the four individually interviewed nurses (II1, II2, II3, and II4) also indicated the lack of computer devices since there was not a computer for each person, which created an unspoken hierarchy of computer ownership with nurses at the bottom. To avoid queuing, nurses had to borrow one from doctors or nursing practitioners. A focus group described:

*E4: When you are waiting, queuing for [the computer] for a while, you are anxious many things are still undone … If necessary, we need to ask [doctors or nursing practitioners] for the computer.*

*E3: We have to be very observant and go to ask [or] if you have a good relationship with a certain NP (nursing practitioner), just use hers.*

(Nurses. Focus Group E. Medical Ward)

Another nurse said

*Each nursing practitioner has her own computer. Even though they already log off the system, you can’t use it as you wish.*

(Nurse C1. Focus Group C. Surgical Ward)

Some participants especially expressed a sense of helplessness about situations when they had urgent needs but no computer available. For instance, focus group participants calculated the number of computers for doctors and nursing practitioners as ten in total, but staff nurses only had one. Also, doctors and nursing practitioners had a higher computer use priority. There seemed no solution given the lack of resources for nurses:

*We nurses only got one computer. Sometimes we hurried to get something for patients, but you can’t do anything about it.*

(Nurse E3. Focus Group E. Medical Ward)

Interestingly, a focus group mentioned that computers were available during the lunch time. So, for non-urgent work they would re-organize their schedule to avoid wasting time queuing for computers or borrowing computers from colleagues, which they disliked doing:

*If you can wait for the lunch time, they [doctors and nursing practitioners] have a lunch break. So you can use that time to check the computerized prescriptions, etc. [and] do things together.*

(Nurse E4. Focus Group E. Medical Ward)

### 4.3. Troubleshooting

Many nurses explained that they would troubleshoot by shutting down and restarting devices before they contacted the informatics staff, but it was not infallible. For example, a nurse said that sometimes shutting down a printer for a while and then restarting it did work (Nurse D1, Pediatric Ward), especially when a network disconnection caused the printer malfunction (Nurse A4, Medica Intensive Care Unit).

Some nurses perceived telephone technical support as an inefficient troubleshooting method, since following the informatics staff’s directions and coping with computer devices was arduous as their different professional backgrounds made understanding each other difficult:

*B4: We spent a long time communicating with the informatics staff by phone. He told us what to do. After a while, he rang back to check what happened on the system. The process repeated and repeated again.*

*B5: He couldn’t realize our problems.*

*B4: Exactly. We could only report what we saw, but it may not be the key part.*

*B5: We could only tell him what we could understand.*

*B1: Their and our specialities are different. It is hard … usually the communication left us disappointed.*

(Nurses. Focus Group B. Surgical Intensive Care Unit)

Consequently, similar to the temporary solution of borrowing medications from other patients, nurses would borrow printers located in other wards as a speedy alternative to tackling printer problems whilst maintaining patient care. For example, a focus group described printing documents on another ward:

*A5: During the night shift, [the informatics support team] usually has only one member on duty. Sometimes, he doesn’t know how to solve the problem. So we would print documents using the printer in our opposite ward.*

*A4: You can choose your printer’s location through the system.*

(Nurses. Focus Group A. Medical Intensive Care Unit)

In summary, although the effectiveness and efficiency of HIS employed in healthcare and management is well documented, the above comments reveal how computer problems had negative impacts on nursing care delivery. The case study hospital had established official coping procedures, which were perceived as time-consuming if applied in sudden, temporary computer-related downtime situations. As meeting patient needs definitely was the first priority, nurses developed rapid “work-rounds” that prevented technical breakdowns delaying patient services. Meanwhile, owing to limited technical support, nurses described their system maintenance work when facing computer-related technical breakdowns. One individually interviewed nurse (Nurse II3 Medical Ward) said: “[We] have to have the manpower to sort it out.” Consequently, nurses are caring for patients and computers.

## 5. Discussions: Unintended Consequences of Computerization

Invisible effort, intangible coordination and troubleshooting are identified as nurses’ coping strategies for computer-related malfunctions and mitigating solutions for the inconvenience they cause. This section discusses the findings based on Wadel’s [59] concept of hidden work. Furthermore, Orlikowski’s [60] concept of duality of technology is employed to explain the fundamental causes of hidden work resulting from HIS.

The concept of hidden work [59] has broadened the concept of work. Wadel defines hidden work as unrecognized activities that have the major characteristics of work but are not called work. They are contingent, indirect efforts vaguely linking with formal work and occurring in everyday life. Due to these attributes, hidden work is easily regarded as a natural progression of events rather than work. Meanwhile, many activities directly related to the production of socially valued goods and services are widely recognized as work, Wadel [59] argues that it is necessary to give these unrecognized everyday activities both active and functional notions. Wadel’s wider concept of work was cited in the study of patient–patient interaction [61] and a study that theorizes the knowledge used by nurses in patient care [62].

Here, Wadel’s concept is helpful to understand the characteristics, value, and functions of the nurses’ coping strategies for computer-related malfunctions. These tasks seem vague, trivial, and difficult to quantify, but are connected with patient care service provision. Participating nurses carried them out naturally as part of their everyday clinical practice whilst regarding direct patient care as their formal work and describing their caring for computers as “trivial matters” that are not considered as work. Such activities as context-related contingencies were unrecognized, unrecorded, and unrewarded, which may contribute to their being difficult to pick up.

In order to further explain the fundamental causes of hidden work resulting from HIS, Orlikowski’s [60] concept of duality of technology is employed. Orlikowski [60] proposes a theoretical model, called the Structurational Model of Technology, to examine the interaction between technology and organizations and has applied it to different kinds of technologies in organizations [63,64,65,66,67]. The key idea of the dualistic view is that technology both enables and constrains human action, which facilitates the performance of certain kinds of work and hence constrains the performance by facilitating it in a particular manner. Orlikowski stresses that the dual influence of the intended and unintended consequences of technology tends to be suppressed by emphasizing a single view of technology and concealing the others in organizations.

The concept of duality of technology is helpful to explain the findings regarding the impact of computerization. Effectiveness and efficiency are the intended consequences of the introduction of HIS, yet the identified activities of managing computer-related technological breakdowns are the unintended consequences that constrain nurses’ professional performance. It regards computers as “a double-edged sword” in relation to nurses’ clinical work. Furthermore, the delay in patient care possibly leads to health deterioration of patients’ conditions. For example, Nurse B5 raised the issue of computerized prescription of blood transfusion therapy.

In their role as system end-users, how nurses perceived computerization and its effects on their work echoes Orlikowski’s concept of the duality of technology. Interestingly, a member of the informatics staff (Software Engineer IT2) raised a reflective question during the interview process: “Does the nursing staff feel that computerization is a help or a burden to them?” The dual influences of HIS on nurses’ performance are identified in the study.

As reviewed in the introductory section, promoting HIS is a worldwide trend. However, as healthcare practice becomes more reliant on computerized information systems, it is also vulnerable to technological breakdowns, such as slow computer-response time and system downtime that affect nurses’ workflow [68,69,70,71,72,73,74], in accord with this study. This indicates that as HIS have led to healthcare service provision that is increasingly dependent on technology, computer-related malfunctions have come to have a negative impact on nurses’ clinical work.

The main research results concern invisible effort, intangible coordination, and troubleshooting, which correspond to nurses’ hidden work as examined in several studies, including queuing for the computer [71,75,76,77], troubleshooting [72], duplication [71,73,78], increased computer time [69,74,75,79], and feeling distanced from patients [70,71,74].

These findings identified nurses’ perceptions of computer technology and the invisible activities resulting from computerization, which are consistent with this study. However, the constraining features and unintended consequences of computer technology are often omitted when promoting HIS in healthcare settings. It also implies that the healthcare organizations may favor computerization in healthcare provision, the so-called pro-innovation bias [80], which requires that an innovation is to be adopted rapidly throughout an organization and should be neither re-invented nor rejected. This stance may focus the computerization process on the nurses’ acceptance of computer technology and consequently lead to healthcare organizations highlighting the intended benefits whilst suppressing the unintended consequences.

Additionally, while Wadel’s social construction analysis of work tends to assume that work is either paid or unpaid; this study develops the concept of “extra work” to highlight work that is hidden. After computerization, the roles of front-line nurses are not only care provision, but have been extended to include responsibility for the HIS user interface. Hence, it is argued that for nurses, patients and computers have become co-clients. There are two reasons for this. Firstly, the responsibility for invisible activities such as coping with technical breakdown on -site falls mainly on nurses rather than those stakeholders who make decisions—managers or the informatics staff. In other words, the constraining features of HIS fall mainly on front-line nurses’ shoulders. Secondly, when examining the value and function of these unintended activities, it appears that hidden work is extra work and also an unintended extra cost. While HIS introduction appears more efficient on the surface, the existence of hidden work means less time for patients. These invisible activities cause nurses extra work and an increased workload to the detriment of direct patient care, thus adding unseen costs to healthcare service systems.

There are several limitations to the study. First, gaining information regarding HIS was restricted. Due to the patient data protection purposes and the issue of intellectual property, the researcher had no access to the HIS of the case study hospital or to review the step-by-step manuals. Second, recruiting front-line nurses for focus groups was more difficult than expected. The reasons seemed to be that they were unfamiliar with the qualitative research method, and attending the focus group discussion was more time-consuming and not as flexible as answering questionnaires to meet their busy schedules. Third, the findings were from a cross-sectional single case study in Taiwan, which restricted the findings to a certain time frame and location. The technology of HIS itself has been evolving internationally since the study has been conducted, nevertheless this study intends to focus on the unintended effects of HIS on nurses’ role and clinical practice in a context of a major East Asian medical center in the era of computerization. Although the results may not be completely generalizable, they can provide a better understanding of nurses’ practical experiences by offering nurses’ perceptions regarding the effect of HIS implementation on their role and practice, thereby providing a reflective understanding of human reliance on technology.

## 6. Conclusions

Promoting the worldwide application of HIS in healthcare services is supported by extensive literature suggesting their potential for enhancing quality of care and management. The research findings show the context of computer-related downtime from the nurses’ perspectives and how they developed coping strategies. The concepts of hidden work and duality of technology may illuminate the nature of these activities. It is revealed that these seemingly trivial and invisible coping activities, like a small cog in a large machine, are significant in maintaining hospital functions. Moreover, as healthcare services are heavily reliant on technology, computer downtime is a prominent issue that could have potential to negatively affect nurses’ clinical work. These additional burdens fall on front-line nurses, thereby consuming nursing time and taking them away from patients whilst making computers and patients co-clients. Although effectiveness and efficiency are the intended consequences of HIS implementation, managing computer-related malfunctions is an unintended influence on nurses’ professional activities. It is argued that in the era of computerization, the role of front-line nurses is not only providing patient care services, but also caring for computers, meaning that patients and computers have become nurses’ co-clients, accompanied with a costly, but invisible price. Further research on the influence of HIS implementation is needed. Studies covering intended and unintended issues of HIS implementation, or focusing on user involvement in information system development are recommended.

## Figures and Tables

**Table 1:** Informatics staff participants (*n* = 3).

ID	Position	Role
IT1	Division leader	Supervision
IT2	Software engineer	In charge of the nursing information system
IT3	Software engineer	In charge of the electronic prescribing system

**Table 2:** Recruitment results of focus groups and in-depth interviews.

Ward	Number of Staff Approached	Number of Participants	
		Focus Group	Individual Interview
Medical Intensive Care Unit-2	54	7 (Group A)	1 (II1)
Surgical Intensive Care Unit-1	59	5 (Group B)	0
Surgical Ward-1	23	3 (Group C)	0
Surgical Ward-2	26	0	0
Paediatric Ward-1 and 2	32	6 (Group D)	0
Medical Ward-1	27	4 (Group E)	0
Medical Ward-2	32	0	0
Medical Ward-3	26	0	2 (II2, II3)
Medical Ward-4	29	0	0
Medical Ward-5	22	0	1 (II4)
Medical Ward-6	29	0	0
Subtotal	359	25	4

**Table 3:** Demographic data for nurse participants (*n* = 29).

	Number	Percentage (%)
Age (years)		
20–24	3	10.5
25–29	16	55
30–34	6	21
35–39	3	10.5
missing	1	3
Gender		
female	28	97
male	1	3
Ethnicity		
native	28	97
foreign	1	3
Educational status		
5 year diploma	7	24
Bachelor/RN to BSN	21	73
Master’s degree	1	3
Seniority		
from 1 to 2 years	4	14
from 3 to 5 years	13	45
from 6 to 10 years	8	27
from 11 to 15 years	2	7
from 16 to 20 years	2	7
Nursing grade		
N0	1	3
N1	10	35
N2	8	28
N3	9	31
N4	0	0
missing	1	3

## Data Availability

The data presented in this study are available within the article.
